# Effectiveness of a comprehensive interactive eHealth intervention on patient-reported and clinical outcomes in patients with an implantable cardioverter defibrillator [ACQUIRE-ICD trial]: study protocol of a national Danish randomised controlled trial

**DOI:** 10.1186/s12872-018-0872-7

**Published:** 2018-07-03

**Authors:** Susanne S. Pedersen, Søren J. Skovbakke, Uffe K. Wiil, Thomas Schmidt, Rene dePont Christensen, Carl J. Brandt, Jan Sørensen, Michael Vinther, Charlotte E. Larroudé, Thomas M. Melchior, Sam Riahi, Kim G. E. Smolderen, John A. Spertus, Jens B. Johansen, Jens C. Nielsen

**Affiliations:** 1 Department of Psychology, University of Southern Denmark, Campusvej 55, DK-5230 Odense M, Denmark; 2 Department of Cardiology, Odense University Hospital, Odense, Denmark; 3 OPEN, Odense Patient data Explorative Networ, Odense University Hospital, Odense, Denmark; 4 The Maersk Mc-Kinney Moller Institute, University of Southern Denmark, Odense, Denmark; 5 Research Unit of General Practice, Odense, Denmark; 6 Danish Center for Health Economics (DaCHE), University of Southern Denmark, Odense, Denmark; 7 Department of Cardiology B, Rigshospitalet, Copenhagen, Denmark; 8 Department of Cardiology, Copenhagen University Hospital, Gentofte, Denmark; 9 Department of Cardiology, Zealand University Hospital, Roskilde, Denmark; 10 Department of Cardiology, Aalborg University Hospital, Aalborg, Denmark; 11 Saint Luke’s Mid America Heart Institute and the University of Missouri-Kansas City, Kansas City, MO USA; 12 Department of Cardiology, Aarhus University Hospital, Aarhus, Denmark

**Keywords:** Anxiety, Depression, Device acceptance, eHealth intervention, Heart disease, Implantable cardioverter defibrillator, Patient-centered tools, Quality of life

## Abstract

**Background:**

In Denmark and other countries, there has been a shift in the management of patients with an implantable cardioverter defibrillator (ICD) with remote device monitoring largely replacing in-hospital visits. Less patient-nurse and patient-physician interaction may lead to gaps in patients’ quality of care and impede patients’ adaptation to living successfully with the ICD. A comprehensive eHealth intervention that include goal-setting, monitoring of symptoms of depression, anxiety, and quality of life, psychological treatment, information provision, supportive tools, online dialogues with nursing staff and access to an online community network, may help fill these gaps and be particularly beneficial to patients who suffer from anxiety and depression. This study will evaluate the effectiveness of the ACQUIRE-ICD care innovation, a comprehensive and interactive eHealth intervention, on patient-reported and clinical outcomes.

**Methods:**

The ACQUIRE-ICD study is a multicenter, prospective, two-arm, unblinded randomised controlled superiority trial that will enroll 478 patients implanted with a first-time ICD or ICD with cardiac synchronisation therapy (CRT-D) from the six implanting centers in Denmark. The trial will evaluate the clinical effectiveness and cost-effectiveness of the ACQUIRE-ICD care innovation, as add-on to usual care compared with usual care alone. The primary endpoint, device acceptance, assessed with the Florida Patient Acceptance Survey, is evaluated at 12 months’ post implant. Secondary endpoints, evaluated at 12 and 24 months’ post implant, include patient-reported outcomes, return to work, time to first ICD therapy and first hospitalisation, mortality and cost-effectiveness.

**Discussion:**

The effectiveness of a comprehensive and interactive eHealth intervention that relies on patient-centred and personalised tools offered via a web-based platform targeted to patients with an ICD has not been assessed so far. The ACQUIRE-ICD care innovation promotes and facilitates that patients become active participants in the management of their disease, and as such addresses the need for a more patient-centered disease-management approach. If the care innovation proves to be beneficial to patients, it may not only increase patient empowerment and quality of life but also free up time for clinicians to care for more patients.

**Trial registration:**

The trial has been registered on https://clinicaltrials.gov/ct2/show/NCT02976961 on November 30, 2016 with registration number [NCT02976961].

## Background

Implantable cardioverter defibrillator (ICD) therapy is the first-line of treatment for the primary and secondary prevention of sudden cardiac death [1]. Since the early primary and secondary prevention trials were published in the 1990’s, demonstrating risk reductions in mortality ranging from 26% and higher, depending on the indication and sub-population [2, 3], the implantation rate has increased substantially. The implantation rate now seems to have stabilised in Western and Northern European countries, while the implantation rate has continued to rise in Eastern European countries [4]. In 2016, the mean number of ICD implantations in Europe was 101 per million inhabitants [4].

The majority of patients implanted with an ICD do well [5], despite ICD therapy being associated with risk of procedural and device-related complications (e.g. infection, lead dislocation, device and hardware malfunctioning, and inappropriate shocks). However, a subset of patients (20%) have difficulties with psychological adjustment post implant and report significant levels of anxiety and depression and poor quality of life (QoL) [6, 7]. Poor patient-reported outcomes may in part be attributed to shocks and fear of shocks [8, 9], which may lead to avoidance behaviours, a sedentary lifestyle, and sexual problems. [10] However, other factors may play an equal or larger role than ICD-related aspects. These include the psychological profile of the patient (e.g. Type D personality [11, 12], lack of optimism [13]), illness perceptions [14], treatment expectations [15], and the underlying disease (e.g. symptomatic heart failure) [16]. Managing patients’ psychological morbidity, although important, is often ignored, even though depression and anxiety have been associated with increased risk of mortality and in some studies with ventricular tachyarrthmias despite state-of-the-art treatment with the ICD [17–19].

In the last decade, we have witnessed a change in the management of ICD patients, with face-to-face outpatient visits largely being replaced by remote monitoring [20], although variability exists between countries. Remote monitoring enables the ICD clinic to detect abnormal heart rhythms and problems with the device faster, with demonstrated clinical and economic advantages [21], although we know less about its impact on patients’ QoL. [22, 23] Despite the convincing clinical and economic advantages this may have led to unrecognised gaps in patients’ quality of care [24]. Due to less patient-nurse and patient-physician interactions, it has become more difficult to identify vulnerable patients who need additional support and follow-up. This is further complicated by the fact that in Denmark and many other countries across the world, patients are not screened for psychological morbidity as part of clinical cardiology practice nor is any treatment for psychological morbidity available in the hospital setting. Patients may also be hesitant to contact the clinic outside of scheduled visits in order not to burden the care team. When examining patients’ preferences and needs for care options that are not part of standard practice, the majority of patients with ICD indicated that they would have preferred to receive continuous feedback via remote monitoring and also to have more interaction with theire care team [24]. Taken together, this may impede the transition to living successfully with the ICD for patients who have difficulties with psychological adjustment after implant.

Intervention trials targeting symptoms of depression and anxiety in the ICD population have largely used cognitive behavioral therapy, relaxation and mindfulness techniques, supplemented with telephone support, education and exercise, and online therapies, as single, one-time interventions [25, 26]. Although some of these have shown promise, with the largest effects found for cognitive behavioral therapy, the majority have been plagued by methodological shortcomings and included relatively small sample sizes [27–29]. The complexity of the issues that patients with an ICD deal with may warrant a more comprehensive intervention that is more patient-tailored and facilitates patient empowerment. As indicated by others, there is a need to continue to search for successful care modalities in order to ensure that patients are able to live the best possible life with their device and disease [30]. This requires “…*a shift from individual blame toward an empowerment and systems approach that considers the big picture*” [31].

One strategy for meeting these needs are asynchronous, web-based strategies. Using an eHealth approach that enables patients to interact at a time and place of their convenience may have distinct advantages, in particular for patients who have to return to work after implantation. Hence, to address these gaps in evidence and clinical practice, we designed the ACQUIRE-ICD study (A personalised and interactive web-based health care innovation to **A**dvan**C**e the **QU**al**I**ty of life and ca**RE** of patients with an **I**mplantable **C**ardioverter **D**efibrillator). A full description of the intervention is described in the methods. ACQUIRE-ICD will formally evaluate the effectiveness of the comprehensive and interactive ACQUIRE-ICD eHealth intervention on patient-reported and clinical outcomes and the cost-effectiveness as add-on to usual care as compared to usual care alone.

### Objectives

#### Primary objective

The primary objective is to investigate the effectiveness of the comprehensive and interactive ACQUIRE-ICD eHealth intervention as add-on to usual care as compared to usual care alone on device acceptance, as measured by the Florida Patient Acceptance Scale, at 12 months post implant (the end of the intervention).

#### Secondary objectives

The secondary objectives are to investigate the effectiveness of the ACQUIRE-ICD eHealth intervention on health status (generic and disease-specific), patient empowerment, ICD concerns, anxiety, depression, return to work, time to first ICD therapy defined as anti-tachycardia pacing (ATP) or shock therapy, time to first hospitalisation due to a cardiac cause, mortality, and cost-effectiveness at 12 and 24 months post implant.

## Methods

### Study design

ACQUIRE-ICD is a multicenter, prospective, two-arm, unblinded randomised controlled superiority trial that will enroll patients receiving their first ICD or CRT-D from the six ICD implanting centers in Denmark: Odense University Hospital; Aarhus University Hospital; Copenhagen University Hospital (Rigshospitalet); Copenhagen University Hospital (Gentofte); Aalborg University Hospital; Zealand University Hospital (Roskilde).

### Subjects and eligibility

**Fig. 1:**
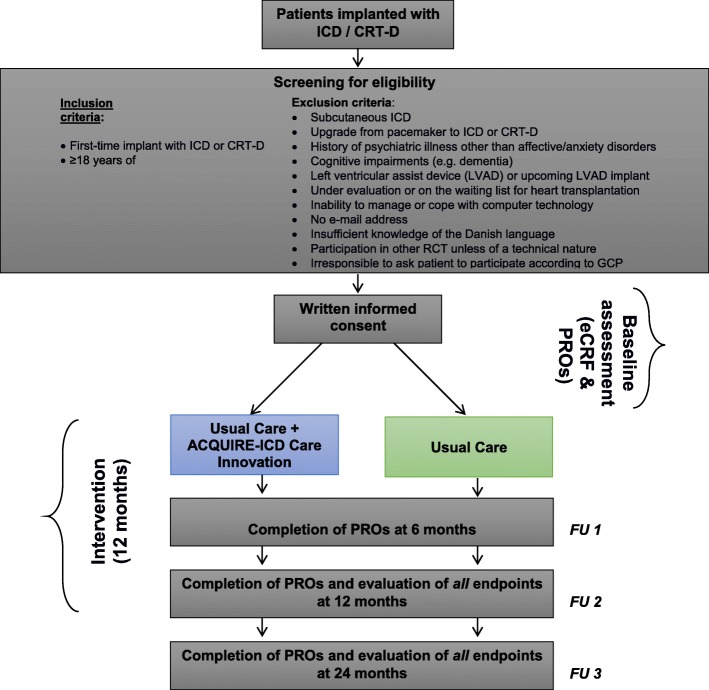
Design and flowchart of patient inclusion and follow-up

A sample of 478 patients will be recruited consecutively from the 6 participating centers. Patients are eligible to participate if they fulfill all of the inclusion criteria and none of the exclusion criteria, as indicated in Fig. 1. The CONSORT guidelines for a parallel-group randomised controlled trial will be used when reporting on the results of the trial [32].

### Patient recruitment

Patients will be asked if they wish to participate in the study while hospitalised for their ICD implantation. After screening for in- and exclusion criteria, eligible patients will receive written and oral information about the study and asked to sign an informed consent form, if they agree to participate. The baseline questionnaire is sent as a link to their personal email and answered online in the Research Electronic Data Capture (REDCap) database, either in hospital or at home and between 48 h pre-implantation and 2 weeks post implantation. Once patients have the ICD implanted and completed the baseline questionnaire, they will be allocated to the interactive web-based care innovation or usual care, with the use of the random allocation sequence. Patients in the intervention arm are given a 45-min introduction, either face-to-face during hospitalisation or via telephone in their home setting, how to access and use the web-based platform. To ensure that there are no technical problems and that patients are able to use the platform, within the first week, they are asked to write a message via the platform to the nurse assigned to them from the implanting hospital.

### Study endpoints

#### Primary endpoint

The primary endpoint is patient device acceptance measured with the Florida Patient Acceptance Survey [33, 34], evaluated at 12 months’ post implant.

#### Secondary endpoints

Secondary endpoints include health status (generic and disease-specific), patient empowerment, ICD concerns, anxiety, depression, return to work, time to first ICD therapy defined as ATP or shock, time to first hospitalisation due to a cardiac cause, mortality, resource use and costs related to the intervention, additional healthcare resource use and costs during the observation period, and cost-effectiveness. All secondary endpoints are evaluated at 12 and 24 months’ post implant. Primary and secondary outcomes are presented in Table 1.

**Table 1:** Primary and secondary endpoints

Primary outcome *[evaluated at 12 months’ post implant]* • Device acceptance (FPAS) Secondary outcomes *[evaluated at 12 and 24 months’ post implant]* • Health status (SF-12) • Patient empowerment (ICD-EMPOWER) • ICD concerns (ICDC) • Anxiety (GAD-7) • Depression (PHQ-9) • Health status (KCCQ-12; for patients with heart failure only) • Return to work • Time to first ICD therapy defined as anti-tachycardia pacing, cardioversion or shock • Time to first hospitalisation due to a cardiac cause • Mortality • Cost of intervention program • Healthcare costs • Labour market absence • Cost-effectiveness (i.e., incremental cost gained per quality adjusted life year (QALY) and burden on health care professionals (physicians and nurses)^a^	

^a^This will be measured with the EQ-5D-5 L or captured from the patient’s electronic health record (EHR; e.g. number of outpatient visits, admissions, consultations with psychologists), and purpose-designed questions

### Measures

#### Demographic and clinical variables

Information on patients’ demographic and clinical baseline characteristics and follow-up events are captured from patients’ electronic health records (EHRs) and entered into REDCap. This information is registered for all patients, both those who consent to participate, refuse participation and drop out of the study, if they provide written informed consent.

#### Patient-reported measures

An overview of the patient-reported measures and related measures and their time of administration are shown in Table 2. All measures are standardised and validated, although purpose-designed questions or questionnaires have been added if relevant and when no validated measure was available (e.g. in relation to the assessment of cost-effectiveness and patient empowerment).

**Table 2:** Patient-reported outcomes, cost-effectiveness and their assessment*

Construct	Scale acronym	Items	*T*_0_	*T*_6_	*T*_12_	*T*_24_
Health status (generic)	SF-12	12	X	X	X	X
Health status (HF specific)	KCCQ	12	X	X	X	X
Depression	PHQ-9	9	X	X	X	X
Anxiety	GAD-7	7	X	X	X	X
Expectations to ICD treatment	EXPECT-ICD	10	X			
Expectations to use of platform/app	EXPECT-APP	6	X			
Experience with use of platform/app	EXPERIENCE-APP	9		X	X	
ICD patient concerns	ICDC	8	X	X	X	X
ICD empowerment	ICD-EMPOWER	14	X	X	X	X
Illness perceptions	B-IPQ	9 + 1	X			
Adherence	MMAS-8	8	X	X	X	X
Type D personality	DS14	14	X			
Device acceptance	FPAS	18	X	X	X	X
Loneliness	UCLA	3	X	X	X	X
Cost of intervention program	€	Registry	X	X	X	X
Healthcare cost during observation period	€	Registry	X	X	X	X
Labour market absence	€	Registry	X	X	X	X
All-cause and cardiac-related mortality	Deaths	Registry	X	X	X	X
QALY	EQ-5D-5 L + VAS	6	X	X	X	X
Cost-effectiveness/utility (ICER)	€/QALY	–	X	X	X	X
Net-monetary benefit from health care and social perspective	€	-	X	X	X	X

*T*_*0***=**_ Baseline*;* *T*_*6*_ *=* 6 months*;* *T*_*12*_ = 12 months*;* *T*_*24*_ = 24 months

^*^Both patients in the treatment group and usual care group will complete all measures at *T*_*0,*_*T*_*6,*_*T*_*12,*_ and *T*_*24*_

*B-IPQ* Brief Illness Perceptions Questionnaire, *DS14* Type D Scale, *EQ-5D-5 L* EuroQoL, *EXPECT-ICD* Expectations to ICD treatment questionnaire, *EXPECT-APP* Expectations towards use of app, *EXPERIENCE-APP* Experience with use of app, *FPAS* Florida Patient Acceptance Survey, *GAD* Generalised Anxiety Disorder scale, *ICD-EMPOWER* ICD Empowerment Scale, *ICDC* ICD Patients’ Concerns Questionnaire, *KCCQ* Kansas City Cardiomyopathy Questionnaire, *MMAS-8* Morisky Medication Adherence Scale, *PHQ-9* Patient Health Questionnaire, *QALY* quality adjusted life year, *SF-12* Short Form Health Survey, *UCLA* UCLA Loneliness Scale, *VAS* visual analogue scale

#### Cost-effectiveness

In order to assess the effectiveness of the care innovation, a cost-effectiveness/cost-utility analysis will be performed, estimating the incremental costs and effects of the ACQUIRE-ICD care innovation and comparing the two arms. The effect, in terms of changed/improved health status/QoL (i.e., Quality Adjusted Life Years), will be estimated using the EQ-5D. Patients will fill in the EQ-5D questionnaire and the associated VAS at different points in time (at inclusion, at 6-, 12-, and 24-months of follow up). The EQ-5D is a standardised instrument for use as a measure of health outcome developed by health economists for this purpose. The measure consists of a VAS, with patients rating their health on a scale from 0 (worst imaginable health status) to 100 (best imaginable health status), and a descriptive system comprising five questions assessing the following domains: Mobility, self-care, usual activities, pain/discomfort, and anxiety/depression [35]. Each question can be scored as: having no problems (level 1), slight problems (level 2), moderate problems (level 3), severe problems (level 4), and extreme problems (level 5). Subsequently, it is possible to estimate a utility score, a single summary index derived on the basis of the EQ-5D domains, with this information being useful in cost-utility analysis.

Resource use and costs relating to the ACQUIRE-ICD care innovation will be obtained prospectively as costs related to individual participants (variable costs) and the program (fixed costs). Healthcare resource use will be analysed based on data extraction from national health registries for individual participants. The costing period will be from the day of entry into the trial and 12/24 months post implantation. Resource data will include services used in the hospital sector (National Patient Registry – somatic and psychiatry inpatient and outpatient contacts, the Primary Care Registry and Registry of Prescription Medication provided by the primary pharmacies). These resources will be valued using current unit costs (converted to €) based on national sources using tariffs for diagnostic related groups (hospital service), reimbursement fees (primary care service), transaction costs (pharmaceutical services), current salaries, project accounts and expert assessment.

Effect, in terms of changed/increased life expectancy, will be estimated based on extraction of vital status from the National Death Registry. This registry includes date of death and recorded casus of death if that has occurred.

In addition, data from the National registry on Social Transfer Payment (DREAM) will be obtained. This registry will provide data on long-term sickness and reimbursement of lost productivity and provide a measure of the absence from the labour market. This will only be relevant for the period that participants are affiliated with the labour market (i.e. until retirement age). The registry will also provide information about early sickness and voluntary retirement (i.e. before retirement age). Difference between the participant groups will be analysed using logistic or time-to-event regression methods. Additional costs in terms of short-term productivity costs due to absence from the work force (# missed workdays patients; # missed workdays caregivers; # hours care giving to patient) will be obtained through participant questionnaires. Labour market absence will be valued using age and gender stratified by average gross wages obtained from Statistics Denmark.

### Intervention

#### Usual care

Irrespective of the treatment condition that patients are assigned to, all patients will receive usual care after implantation. This includes usual clinical follow-up visits in the outpatient ICD clinic by specialists and nursing staff, their device being monitored remotely, and for relevant patients, follow-up visits to other specialised clinics for e.g. heart failure and arrhythmogenic disorders.

#### Comprehensive interactive ACQUIRE-ICD eHealth intervention

The ACQUIRE-ICD eHealth intervention is delivered via the Liva Healthcare platform, which was developed to help patients manage chronic diseases: https://livahealthcare.com/the-platform/. Liva is browser-based for the health care professionals, with access to the platform and its tools for patients via native solutions for smartphone (IOS and Android), tablet / iPad, or a website application. The platform makes it possible for patients, health care professionals and other stakeholders to access, monitor and update personal health data in a secure ISO 27001 environment. It allows for patients to write personal messages to health care professionals and vice versa, to set targets for behavioural change, to receive push messages to remind them to monitor lifestyle and complete questionnaires, take action if targets are not met, to engage in dialogues with fellow patients (via the platform’s forum), and for health care professionals to support patients with positive reinforcement and multi-media contents (vodcasts, pdf-files, etc.), so that patients can reach their goals [36].

The intervention consists of various components and lasts 12 months. An overview of the components is provided in Table 3 and a more detailed description is provided below.

**Table 3:** Overview of the components of the ACQUIRE-ICD intervention

• Goal-setting for behavioral change • Monthly patient and clinician tracking and monitoring of symptoms of depression (PHQ-8), anxiety (GAD-7) and health status (EQ-VAS) and feedback • Referral for psychological treatment based on value-based cognitive behavioral therapy [*only for patients with a positive screen on depression and/or anxiety*] • Dialogues with nursing staff via the platform [*1–3 months post implant: once a week; 4–12 months: once a month; potential to obtain extra feedback*] • Information provision and education (e.g. on ICD-related topics, anxiety, depression, relaxation training, by means of multi-media contents, such as quizzes, vodcasts of patients and health care professionals, etc.) [examples of vodcasts are available here: https://helbredsprofilen.dk/en] • Forum with online community network with *“patients like me”*	

*EQ-VAS* EuroQoL visual analogue scale, *GAD* Generalised Anxiety Disorder scale, *PHQ* Patient Health Questionnaire

##### Goal-setting for behavioral change

Via the platform, patients can set goals and targets for change, such as exercising more, ensuring good sleep hygiene, eating more healthily etc. The platform provides tailored feedback and delivers positive reinforcement to patients via push messages to encourage them to fulfill their goals. When setting goals and targets for change, the SMART (Specific, Measureable, Achieveable, Realistic and Timebound) criteria are used, in order to keep patients motivated and enhance patient success and outcomes, while also recognising barriers to change, such as depression [37].

##### Completion and monitoring of symptoms of depression and anxiety and health status

Depression and anxiety symptom and health status monitoring was included in the intervention to educate patients about their physical and mental health, as symptoms of depression and anxiety may serve as a barrier to behaviour change and compliance. Without this awareness, patients’ endeavours to change are likely to fail, which may decrease their motivation on the long-term, and influence their QoL and successful disease management. Patients themselves indicate that psychological status is one of the primary reasons for hospital readmission, with unexpectedly few patients pointing to non-compliance as the culprit [31]. Poor QoL and poor patient-rated health status have been shown to predict poor prognosis and mortality in patients with an ICD, despite state-of-the-art treatment [38].

Patients will receive automatic prompts once a month to complete the Patient Health Questionnaire (PHQ-8) [39], the Generalised Anxiety Disorder scale (GAD-7) [40], and the EuroQol visual analogue scale (EQ-VAS) [41] to monitor their symptoms of depression and anxiety and health status, respectively. We chose to use the 8-item version of the PHQ rather than the 9-item version, removing the item on suicidal ideation, as it is not an accurate suicide screen and few cardiac patients endorse this item [39]. The core symptoms of depression as assessed with the PHQ-2 or PHQ-9 predict new onset myocardial infarction and mortality [42]. Following completion of the questionnaires via the platform, the scores are plotted into a graph and added to previously completed scores to generate a graphic summary showing the evolution of scores over time. This allows patients and nursing staff to routinely track patients’ status, flag deteriorations early on that serve as cues for timely action for patients and the ICD care team to discuss whether action needs to be taken. An automatic reminder system is in place if patients do not complete the questionnaires. Prior to the ACQUIRE-ICD trial, we evaluated the experiences of patients and nursing staff with health status monitoring in a feasibility study of patients with health failure. Although not all patients were positive about monitoring, several patients and the nursing staff found it a useful tool and felt that it provided a more true picture of how patients felt [43].

Patients will receive feedback from the nursing staff every time they have completed the questionnaires. If patients experience a decline ≥10 points (minimal clinical importance difference) on EQ-VAS [41], nursing staff will ask patients to reflect on the reasons for this decline, and whether action needs to be taken. If patients have an increased depression or anxiety score on the PHQ-8 or GAD-7 score ≥ 10 (moderate symptoms), nursing staff will refer the patient to the project’s psychologists for online treatment.

##### Online psychological treatment

An online treatment program, based on value-based cognitive behavioral therapy, was specifically developed for the study. The psychologist will contact the patient who is referred by the nursing staff and organise an intake over the phone. During the intake – which is similar to the standard intake when the psychologist meets with the patient face-to-face for the first time – the patient and the psychologist discuss how the patient is doing and what might have contributed to the onset of anxiety or depression. If the patient feels impaired in his / her daily living and consents to start with treatment, the psychologist will initiate treatment that consists of 7 steps: *(i)* ICD and its impact; *(ii)* When one’s life changes; *(iii)* The impact of thoughts on mood and actions; *(iv)* Acceptance and values – living a valuable life; *(v)* How does my daily life looks like?; *(vi)* Find a good routine; *(vii)* To continue to develop and accept stagnation or relapse. If the patient has already contacted or is being seen by a psychologist, the patient will not be offered parallel treatment in the ACQUIRE-ICD study. In order to increase patient compliance with the psychological treatment, patients are offered a phone consultation mid-way (Step 3) and towards the end (Step 7) of the psychological treatment. If patients screen positive for anxiety and / or depression again, the patient will again be referred to the psychologist.

##### Dialogues with nursing staff via the platform

Dialogues via the platform with nursing staff happen asynchronously. The patient can always enter data and see on the platform when the nurse will send a message or respond to the patient’s message. During the first 3 months of the intervention, the nurse will send weekly messages, while in months 4–12, there will be one message a month. Additional messages are allowed on a need-to-basis.

##### Information provision and education

To support behavioural change and to enhance patient motivation and compliance, a toolbox is developed that contains educational multimedia content, such as vodcasts, links, text and pictures, e.g. from *‘Helbredsprofilen’* https://helbredsprofilen.dk/en. The toolbox that was developed for the ACQUIRE-ICD study has a broad focus with topics on ICD treatment and management, self-care management, resources for lifestyle changes and management (e.g. exercise tips), how to set realistic goals, how to keep up motivation, and psychological and social issues, etc. The toolbox also contains quizzes to inform and test patients’ knowledge and educate them about their device, heart disease, psychological aspects and related topics. In order not to overwhelm patients with too much or irrelevant information, nurses will send the contents of the tool box to patients on the times scheduled for contact via the nursing dialogues mentioned above according to a pre-specified plan based on clinical experience and when patients may need the information. These supportive tools can be tailored to the individual needs of patients.

##### Forum with online community network

This feature was included in the intervention, as evidence has shown that online communities with peer-to-peer support from “*patients like me*” that allow for patients to share their stories and experiences is a successful addition to web-based and digital solutions to facilitate behavioural change and to provide social support, as it makes patients feel less alone [44]. The community is part of a mature community with more than 1000 members and will undergo a mitosis after the inclusion of 50 patients to strengthen the domain of ICD patients [45]. As there is a known trade-off between moderation and control on online patient communities and as we wanted to ensure that patients feel free to express and share their thoughts and experiences freely, we decided moderation only by a “watchdog” – the community manager of the project team – who follows the discussions on the forum and redirect the conversation positively if it goes in a less helpful manner [46].

### Sample size calculation

Based on historical data from the Web-based Distress Management Program for Implantable Cardioverter Defibrillator (WEBCARE) patients that evaluated the effectiveness of an online behavioural treatment based on problem-solving principles of cognitive behavioural therapy [47, 48], we assume that a difference in device acceptance, as measured by the Florida Patient Acceptance Survey, of 3 points at 12 months is the minimal clinically relevant difference between treatment groups. We further assume a standard deviation (SD) of 9 points. Targeting the power analysis to these assumptions, with a type I error of 0.05 (two-sided) and a power of 90%, 382 patients need to be evaluable for the primary endpoint. To compensate for loss to follow-up (estimated at 20%), we will aim to randomise a total of 478 patients (239 in each arm).

### Randomisation, blinding and treatment allocation

Patients are randomised in a 1:1 fashion to the ACQUIRE-ICD eHealth intervention plus usual care versus usual care alone, stratifying by center and heart failure symptom severity (New York Heart Association (NYHA) functional class I-II versus III-IV). A random allocation sequence will be generated for each stratum by an independent statistician. The sequence determines the allocation for each subsequent patient. It is not possible to blind patients as to their study condition.

### Ethics and safety considerations

The study will be conducted according to the principles of the Declaration of Helsinki (64th WMA General Assembly, Fortaleza, Brazil, October 2013). The study protocol was submitted to the Regional Committees on Health Research Ethics for Southern Denmark, who indicated that according to Danish law about ethics related to health research (§ 14, 1), ethical committee approval is not required (VEK #S-20160063, June 13, 2016). The Danish Data Protection Agency, via the umbrella scheme in the Region of Southern Denmark (OUH #16/16935, June 21, 2016), has approved the project. Permission from the Danish Health Authority is normally required if information from patients’ EHRs need to be used for specific research projects (health law § 46, 2). However, this is not required if patients provide written consent that they are willing to pass on this information, as is the case for ACQUIRE-ICD. The trial has been registered on https://clinicaltrials.gov/ct2/show/NCT02976961 [NCT02976961].

### Statistical methods

#### General analyses

Data will be analysed according to the modified intention-to-treat principle (i.e., all patients with a 12-month evaluation will be analysed as randomised). Categorical variables will be summarised as n (%), continuous variables as min-max, mean (SD), and median. All summaries will be reported by randomisation group. Kaplan-Meier models will be used to estimate survival functions related to time-to-event endpoints e.g. mortality, stratified by group. Regression analyses, linear, logistic, and Cox proportional hazard will be used as appropriate. The type of analysis will depend on the endpoint in question. The primary analysis will be a linear regression of patients’ scores on the Florida Patient Acceptance Survey at 12 months including randomisation group, baseline score and strata as factors and patients’ Florida Patient Acceptance Survey scores at baseline as covariates. Potential confounders, apart from baseline and stratification variables, identified by clinical consensus, will be included in multivariable analyses when appropriate (e.g. when performing sub-group analyses). Additionally, a mixed effects model will be used to analyse the longitudinal change in mean device acceptance scores on the Florida Patient Acceptance Survey while adjusting for strata and baseline Florida Patient Acceptance Survey scores, incorporating random effects to model the homogeneity within cluster and the correlation within patient across time.

Subgroup analyses are planned for the primary endpoint with respect to: Type of device (CRT-D versus ICD); symptomatic heart failure (NYHA class I-II (mild) versus NYHA class III-IV (severe)); indication (primary versus secondary prevention); sex (female versus male); age (median split); depression status (PHQ score < 9 versus ≥10). All other subgroup analyses are explorative.

#### Cost-effectiveness analyses

The analysis of resource use and costs will be conducted on individual participants’ aggregated cost measures during the observation period (12/24 months) from the inclusion date for each individual participant. Cost data are usually skewed and therefore require special analytical methods. The analysis will use the current standard methodology for cost data (generalised regression assuming logistic link function and residuals with gamma distribution). Other methods will be explored to identify the specifications that provide better fit to the data.

All-cause mortality, survival time, retirement and time to retirement will be estimated from inclusion date until death or censoring using appropriate statistical models (Cox-regression or similar). All-cause and cardiac-related mortality will also be used as measures of effect in the cost-effectiveness analysis.

Quality-adjusted life years (QALYs) will be estimated for each individual participant using all the available data points from the EQ-VAS and EQ-5D instrument. The EQ-5D scores will be converted to utility measures (index ranging from 0 to 1) using the recommended algorithm. Work is currently in progress to establish an updated Danish value set. The analysis will be based on the most updated value set at the time of analysis. Sensitivity analysis will be conducted using different value sets. The QALYs will include the area “under the curve” of the different data points during the observation period or until death.

The cost-effectiveness/cost-utility analysis generates results in terms of incremental cost-effectiveness ratios (ICERs). This analysis will use the data set of individual participants’ monitarised resource use (cost) and QALY. Mixed level difference-in-difference modelling will identify the average incremental cost and effect of the intervention in comparison with the control group (the ICER). By assuming a society treshold value for health benefits (QALYs), the net monetary value of the intervention will be analysed for the whole study population (intention-to-treat and per-protocol) and subgroups of the population. In order to assess the precision, the ICERs sensitivity analysis (relating to structural uncertainty) and bootstrapping methods (relating to statistical/parameter uncertainty) will be performed. Both a short-term and a long-term cost-effectiveness analysis (CEA) will be performed. The short-term CEA will use 12/24 months’ time horizon (equivalent to the two-year follow-up period) based on the data obtained through the trial. The long-term CEA will have a lifetime time horizon and will employ a purpose-built model of health outcomes and resource use. The model will extend both cost and effect data obtained from the trial using appropriate statistical methods. Analyses of the CEA will include probabilistic analyses, sensitivity analyses, and scenario analyses. The main cost-effectiveness results will be described as the incremental cost per QALY gained and net-monetary benefit.

Before release of the data for analyses, the statistician involved in the project will write a detailed statistical analysis plan that will be subject to review and approval by the steering committee. Data will be analysed using STATA and R via OPEN Analyse, which is an analysis environment (platform), where data can be stored and processed securely on a server, which is in compliance with the General Data Protection Regulation Directive 95/46/EC.

### Study status

The study started recruitment in February 2017. By June 2018, 226 patients were recruited and randomised. We expect to have recruited all patients by May 2019 and to have completed the 12 months follow-up on May 2020 and the 24 months follow-up on May 2021.

## Discussion

In the multi-center WEBCARE study that was conducted in the Netherlands, we previously evaluated the effectiveness of a simpler online behavioural treatment that was focused on the treatment of anxiety and depression [47, 48]. To our knowledge, the ACQUIRE-ICD study is the first study to evaluate the effectiveness and cost-effectiveness of a comprehensive interactive eHealth intervention in patients implanted with an ICD or CRT-D. The intervention represents an innovative and sustainable paradigm shift of care for patients with an ICD, as it promotes and facilitates that patients become active participants in the management of their own disease. It addresses the need for a more patient-tailored disease-management approach with care being defined by individual patients’ needs and preferences, and extra resources being allocated to those patients who need it the most. By systematically incorporating the patient perspective and patient tools in routine clinical care, the ACQUIRE-ICD study adheres to the recommendations of the American Institute of Medicine for improving the health care system of the twenty-first century to a system that provides consistent and high-quality care that is patient-centred. [49] As indicated in a recent editorial, a patient-centred approach may be paramount in order to advance the quality of care: *“The practice of medicine has evolved from supposition based to science- and evidence-based, often extrapolating laboratory science to the bedside. However, studies that include patient outcomes are showing, with discomforting frequency, that strategies once considered optimal often fall short of their promise when success is defined as an improvement in what the patient experiences”* [50]. Relying on a patient-centred approach, tools and outcomes may not only lead to greater patient compliance, increase patient treatment satisfaction and wellbeing but also improved prognosis [51, 52].

The current study has some important strengths and some limitations. One of the strengths of the current study relates to the design as a multi-center national study, as all 6 ICD implanting centers in Denmark are participating and recruiting patients. In addition, study endpoints include not only clinical outcomes and cost-effectiveness but also patient-reported outcomes, as advocated both by the American Heart Association [53] and the European Society of Cardiology [54], when evaluating the effects of clinical trials. A limitation is that it is not possible to blind patients to their condition and whether they are randomised to the intervention plus usual care versus usual care alone. Lack of blinding may lead to bias and exaggeration of the effect size [55].

## Conclusion

This article presents the study protocol of the ACQUIRE-ICD study, a national multi-center randomised controlled trial that will evaluate the effectiveness of a comprehensive interactive eHealth intervention on patient-reported and clinical outcomes and the cost-effectiveness as add-on to usual care as compared to usual care alone. The ACQUIRE-ICD care innovation promotes and facilitates that patients become more active participants in the management of their own disease, and as such addresses the need for a more patient-centered disease-management approach.

## Abbreviations

ACQUIRE-ICD: A personalised and interactive web-based health care innovation to **A**dvan**C**e the **Q****U**al**I**ty of life and ca**RE** of patients with an **I**mplantable **C**ardioverter **D**efibrillator
ATP: Anti-tachycardia Pacing
CEA: Cost-effectivness analysis
CONSORT: Consolidated Standards of Reporting Trials
CRT: Cardiac resynchronisation therapy
CRT-D: Implantable cardioverter defibrillator with cardiac synchronisation therapy
EHR: Electronic Health Record
EQ-VAS: EuroQoL visual analogue scale
FPAS: Florida Patient Acceptance Survey
GAD: Generalised Anxiety Disorder scale
GCP: Good Clinical Practice
ICD: Implantable cardioverter defibrillator
ICDC: ICD Patients Concerns Questionnaire
ICERs: Incremental cost-effectiveness ratios
KCCQ: Kansas City Cardiomyopathy Questionnaire
LVAD: Left ventricular assist device
NYHA: New York Heart Association
OPEN: Odense Patient data Explorative Network
OUH: Odense Univeristy Hospital
PHQ: Patient Health Questionnaire
QALY: Quality Adjusted Life Year
QoL: Quality of life
REDCap: Research Electronic Data Capture
SD: Standard Deviation
SF: Short Form Health Survey
SMART: Specific Measureable Achieveable Realistic and Timebound
